# Comparison of Differentiation Pattern and WNT/SHH Signaling in Pluripotent Stem Cells Cultured under Different Conditions

**DOI:** 10.3390/cells10102743

**Published:** 2021-10-14

**Authors:** Barbara Świerczek-Lasek, Damian Dudka, Damian Bauer, Tomasz Czajkowski, Katarzyna Ilach, Władysława Streminska, Agata Kominek, Katarzyna Piwocka, Maria A. Ciemerych, Karolina Archacka

**Affiliations:** 1 Department of Cytology, Institute of Developmental Biology and Biomedical Sciences, Faculty of Biology, University of Warsaw, 02-096 Warsaw, Poland; b.swierczek@biol.uw.edu.pl (B.Ś.-L.); dudka@sas.upenn.edu (D.D.); d.bauer@10g.pl (D.B.); tomczajkowski90@gmail.com (T.C.); kilach@biol.uw.edu.pl (K.I.); krymar@biol.uw.edu.pl (W.S.); ciemerych@biol.uw.edu.pl (M.A.C.); 2 Laboratory of Cytometry, Nencki Institute of Experimental Biology, Polish Academy of Sciences, 02-093 Warsaw, Poland; a.kominek@nencki.edu.pl (A.K.); k.piwocka@nencki.edu.pl (K.P.)

**Keywords:** pluripotent stem cells, culture conditions, differentiation, WNT signaling, SHH signaling

## Abstract

Pluripotent stem cells (PSCs) are characterized by the ability to self-renew as well as undergo multidirectional differentiation. Culture conditions have a pivotal influence on differentiation pattern. In the current study, we compared the fate of mouse PSCs using two culture media: (1) chemically defined, free of animal reagents, and (2) standard one relying on the serum supplementation. Moreover, we assessed the influence of selected regulators (WNTs, SHH) on PSC differentiation. We showed that the differentiation pattern of PSCs cultured in both systems differed significantly: cells cultured in chemically defined medium preferentially underwent ectodermal conversion while their endo- and mesodermal differentiation was limited, contrary to cells cultured in serum-supplemented medium. More efficient ectodermal differentiation of PSCs cultured in chemically defined medium correlated with higher activity of SHH pathway while endodermal and mesodermal conversion of cells cultured in serum-supplemented medium with higher activity of WNT/JNK pathway. However, inhibition of either canonical or noncanonical WNT pathway resulted in the limitation of endo- and mesodermal conversion of PSCs. In addition, blocking WNT secretion led to the inhibition of PSC mesodermal differentiation, confirming the pivotal role of WNT signaling in this process. In contrast, SHH turned out to be an inducer of PSC ectodermal, not mesodermal differentiation.

## 1. Introduction

Pluripotent stem cells (PSCs), such as embryonic stem cells (ESCs) or induced pluripotent stem cells (iPSCs), are characterized by multidirectional differentiation potential and unlimited ability to self-renew. Since their derivation, PSCs serve as an essential model to study early stages of development and cell fate specification. Moreover, they are considered a potential source of cells that could be used as a treatment for degenerative diseases, caused by the lack or dysfunction of cell populations. The results of the first clinical trial conducted using human ESC-derived retinal pigmented epithelium showed that such cells are able to survive in recipient eye and improve sight [1]. However, despite numerous protocols enabling differentiation of PSCs into specific cell types, clinical trials have not been completed so far, since there are several requirements to be met to obtain clinically relevant cell populations. Such protocols would have to match, e.g., the following criteria: (1) short time to derive differentiated cells, (2) relatively easy procedure and (3) lack of animal-derived reagents such as serum, which increase the risk of pathogen transmission. Additional hurdles limiting the potential clinical application of PSC-derivatives are connected with the generation of cells other than desired, as well as insufficient cell differentiation generating the risk of teratoma formation.

Such challenges still accompany numerous attempts regarding PSC differentiation, e.g., the myogenic one. Among strategies tested so far are those based on the overexpression of factors crucial for skeletal muscle development, such as *Pax3* or *Pax7* [2,3] or *MyoD* [4,5]. Although they are characterized by high conversion efficiency, the risk of random integration sites in such cells significantly limits the safety of their application in therapies [6]. Other studies aim at mimicking embryo myogenesis in PSCs cultured in vitro by applying factors crucial for this process, such as FGF2, NOTCH, BMP, IGF-1, or HGF, which play important roles at different phases of cell specification, e.g., mesoderm formation or muscle precursor cell differentiation (summarized in [7,8]). Despite the importance of such protocols, the conversion efficiency varies between PSC lines and other cell types, such as neurons are also formed [9]. Moreover, such procedures are usually laborious and time-consuming [10,11]. Most importantly, many of them include animal-derived serum or cell-conditioned medium [12,13,14]. 

In the current study, we compared the fate of PSCs cultured under different conditions: (1) chemically defined by using medium supplemented with serum replacement (abbreviated SR [15]), devoid of any animal-derived reagents, and (2) standard one relying on the use of serum-supplemented medium [16]. In an attempt to focus on PSC mesodermal differentiation, which must occur before ultimate myogenic differentiation of PSCs, we applied factors involved in mesoderm/myogenesis induction: WNTs and SHH. It is important to underline that instead of analyzing a single WNT, we performed analysis of all known WNTs (19 factors in mammals) in undifferentiated and differentiating PSCs. To do this, we took advantage of numerous reagents available for animal cell analysis and performed experiments using mouse embryonic stem cells (mESCs), which were cultured in the presence of either serum or SR, with the last one being devoid of any animal-derived reagents. 

WNTs can act through numerous signaling pathways with the best known canonical, β-CATENIN-dependent pathway and noncanonical ones, such as pJNK-dependent one [17]. The β-CATENIN-dependent pathway was shown to be important for mesoderm specification in the mouse embryo, directly inducing the expression of *Tbxt*, encoding BRACHYURY, and *Msgn1*, i.e., crucial mesodermal regulators [18,19]. It was also demonstrated to be pivotal in inducing PSC differentiation into mesoderm in media supplemented with serum or under chemically defined conditions [20,21,22]. However, another line of evidence showed that canonical WNT signaling is involved in maintaining a pluripotent state [23,24,25]. Despite recent progress and its well-documented role in cardiogenesis, little is known about noncanonical WNT signaling in the early stages of embryo development and PSC differentiation [26,27]. 

The SHH signaling pathway, which acts together with WNT1 and WNT3 in muscle precursor specification during embryogenesis, is mediated by GLI transcription effectors [28]. The role of SHH in PSC differentiation into mesoderm has been addressed previously, but the results obtained so far are contradictory: in one study, this factor was shown to promote ESC differentiation into ectoderm and motor neurons [29], while another line of evidence suggests that it can promote mesodermal differentiation of pluripotent embryonic carcinoma cells [30]. 

In our experiments, to induce ESC differentiation, we used broadly applied ESC culture in aggregates in suspension or in monolayer. In each case, we used a medium lacking leukemia inhibitory factor (LIF), i.e., the factor enabling self-renewal of mouse PSCs [31]. Cells building embryoid bodies (EBs), spherical aggregates formed by PSCs cultured in suspension, differentiate into all three germ layers: ecto-, endo-, and mesoderm [32]. We carefully followed and compared this process in EBs cultured in the presence of either serum or SR, i.e., under chemically defined conditions. We analyzed EB morphology, cell cycle progression, and expression of pluripotency and differentiation markers. To follow subsequent stages of PSC differentiation, i.e., generation of germ layer derivatives, we also analyzed EB outgrowths. With full awareness of possible differences between features and fate of animal and human cells, we addressed whether WNTs or SHH can influence PSC differentiation under both culture conditions with special emphasis on mesodermal conversion.

## 2. Materials and Methods

### 2.1. Cell Culture

Mouse ESCs constitutively expressing histone H2B fused with GFP were provided by Prof. Kat Hadjantonakis, Memorial Sloan Kettering Cancer Center in New York [33]. ESCs were expanded on a feeder layer (inactivated mouse embryonic fibroblasts, MEFs) and cultured in Knockout DMEM (Thermo Fisher Scientific, Waltham, MA, USA) supplemented with 15% ESC-qualified fetal calf serum (FCS, Thermo Fisher Scientific), 0.1 mM nonessential amino acids (Thermo Fisher Scientific), two mM L-glutamine (Thermo Fisher Scientific), 0.1 mM β-mercaptoethanol (Sigma Aldrich, St. Louis, MO, USA), 50 U/mL penicillin (Thermo Fisher Scientific), 50 µg/mL streptomycin (Thermo Fisher Scientific), and 500 U/mL leukemia inhibitory factor (LIF; Chemicon, MerckMillipore, Warsaw, Poland). Before each experiment, the cells were passaged twice.

### 2.2. In Vitro Differentiation of ESCs

ESCs were differentiated either in monolayer culture or in suspension in embryoid bodies (EBs), and then in outgrowths derived from EBs (EBOs). Two types of culture media, both lacking LIF, were used. To form EBs, the ESCs were separated from MEFs using the pre-plating method. Briefly, ESCs and MEFs were plated in gelatin-coated dishes and incubated at 37 °C for 20 min. The procedure was repeated thrice and unattached ESCs remaining in the suspension were collected. Next, 800 ESCs were suspended in 30 µL drops of medium supplemented either with 15% Serum Replacement (SR, Thermo Fisher Scientific) or 15% ESC-qualified FCS. Subsequently, SR medium and chemically defined medium were used interchangeably. Drops were placed onto the covers of culture dishes filled with phosphate-buffered saline (PBS). On day 2 of culture in so-called hanging drops, EBs (referred to as EB2) were transferred to low-adhesive culture dishes (Medlab, Raszyn, Poland) and cultured in suspension for a further three or five days (referred to as EB5 or EB7, respectively). For further differentiation EB2, EB5 and EB7 were transferred to the adhesive culture plates coated with 3% gelatin where they formed outgrowths, referred to as EBOs (EB2, EB5 or EB7-derived outgrowths are referred to as EB2O, EB5O, EB7O, respectively). EBOs were cultured for additional 7 days. The schematic outlines of experiments are shown in Supplementary Figure S1.

### 2.3. WNT11 Treatment

ESCs cultured in monolayer or EB2 cultured in medium supplemented with SR (without LIF) were treated with recombinant WNT11 protein (R&D Systems, Minneapolis, MN, USA) at the final concentration of 250 ng/mL for 48 h and collected for further analyses (referred to as ESC(2) and EB2(2), respectively). Additionally, outgrowths were derived from control and treated EB2 and cultured for 7 days, and collected for further analyses (referred to as EB2(2)O). The schematic outline of experiments is shown in Supplementary Figure S1.

### 2.4. Modulation of WNT Signaling Pathways

To inhibit secretion of all WNTs or block either canonical or noncanonical WNT pathway EBs were cultured either in medium supplemented with 15% SR or 15% ESC-qualified FCS in the presence of either 5 µM IWP-2 (inhibitor of WNT secretion; Stem Cell Technologies, Vancouver, Canada), or 5 µM IWR-1 (canonical WNT pathway inhibitor; Stem Cell Technologies) or SP600125 (noncanonical WNT pathway inhibitor, JNK inhibitor; Stem Cell Technologies) for 2, 5 or 7 days. Next, the obtained EB2, EB5, or EB7 were collected for analyses. 

For the activation of canonical or noncanonical WNT signaling, EBs were cultured in the presence of either 5 µM CHIR99021 (Tocris, Bristol, United Kingdom) or 0.5 µM anisomycin (Tocris), respectively. EBs were cultured for 2, 5, or 7 days and then collected for analyses. The schematic outline of experiments is shown in Supplementary Figure S1.

For siRNA knockdown experiments, undifferentiated ESCs, separated from MEFs, were seeded in gelatin-coated dishes in ESC culture medium, supplemented with LIF, as described above. After reaching approximately 80% confluence, ESCs were transfected with Lipofectamine^®^ RNAiMAX Transfection Reagent (Thermo Fisher Scientific) with 25 pmol Silencer Pre-designed siRNA complementary to *Fzd4* or *Ror2* mRNAs, according to the manufacturer’s instruction. After 48 h, EBs were formed and cultured for 2, 5 or 7 days and then collected for analyses.

### 2.5. SHH Treatment

ESCs cultured in monolayer or EB2 [further referred to as ESC(2) or EB2(2), respectively] were cultured for 48h in medium supplemented with either 15% SR or 15% ESC-qualified FCS and recombinant SHH protein (Thermo Fisher Scientific ) at the final concentration of 200 ng/mL. Additionally, outgrowths were derived from control and treated EB2, cultured for 7 days, and then collected for further analyses [referred to as EB2(2)O]. The schematic outline of experiments is shown in Supplementary Figure S1.

### 2.6. RNA Isolation and qPCR Analysis

Total RNA was isolated from undifferentiated ESCs, EBs, and EBOs cultured in medium supplemented either with 15% SR or 15% ESC-qualified FCS, and 13.5-day-old mouse embryos (which served as a reference sample) using a High Pure RNA Isolation Kit (Roche, Basel, Switzerland) and transcribed to cDNA with RevertAid First Strand cDNA Synthesis Kit (Thermo Fisher Scientific), according to the manufacturer’s protocols. qPCR analysis was performed using specific TaqMan^®^ assays (Thermo Fisher Scientific; listed in Supplementary Materials Table S1), TaqMan Gene Expression Master Mix (Thermo Fisher Scientific) and LightCycler 96 instrument (Roche). Data was collected and analyzed with LightCycler 96 SW1.1 software (Roche). For each analysis at least three independent experiments were performed. ΔΔCq analysis was performed according to Livak and Schmittgen [34] with 13.5-day-old mouse embryo serving as a reference sample and *β-actin* as a reference gene. The mean ΔCq for the reference sample calculated from at least six or more replicates is shown on the graphs. 

### 2.7. Flow Cytometry Analysis

Undifferentiated ESCs were trypsinized and purified from MEFs (as described previously), and EBs cultured in medium supplemented with either 15% SR or 15% ESC-qualified FCS were disaggregated using Enzyme-Free Hanks’-based Cell Dissociation Buffer (Thermo Fisher Scientific) for 5 min at 37 °C. For cell cycle analysis, the obtained cells were washed with PBS and fixed with cold 99.6% ethanol. Fixed cells were incubated with 10 µg/mL ribonuclease (Sigma Aldrich) and 50 µg/mL propidium iodide (Sigma Aldrich) for 30 min at 37 °C. After incubation, the cells were resuspended in PBS and analyzed with LSRFortessa cytometer (BD Biosciences, Franklin Lakes, NJ, USA) and FACSDiva 6.2 software.

For protein detection, single-cell suspension was subsequently fixed, permeabilized, and stained using Transcription Factor Buffer Set (BD Biosciences), according to the manufacturer’s protocol. Next, cells were incubated with primary antibodies on ice for 30 min. The following antibodies were used: anti-NANOG (diluted 1:100; CosmoBio, Tokyo, Japan, RCAB002P-F), anti-SOX2 (diluted 1:1000, Abcam, Cambridge, United Kingdom, ab97959), anti-FZD5 (diluted 1:100, LifeSpan Bioscience, Seattle, WA, USA, LS-A4278-50), anti-FZD7 (diluted 1:100, LifeSpan Bioscience LS-C6916-100), anti-PTC1 (diluted 1:50, Santa Cruz, Dallas, TX, USA, sc-6149) or anti-PTC2 (diluted 1:50, Novus Biologicals, Centennial, CO, USA, NB200-119). Subsequently, the cells were incubated with appropriate secondary antibody conjugated with Alexa Fluor 647 (diluted 1:100, Thermo Fisher Scientific) at 4 °C for 30 min. Cells were analyzed with LSRFortessa cytometer (BD Biosciences) and FACSDiva 6.2 software.

### 2.8. Immunolocalization

For whole-mount immunolocalization of EBs, aggregates cultured in medium supplemented with either 15% SR or 15% ESC-qualified FCS were plated on 10% growth factor-depleted Matrigel (BD Biosciences) and fixed after 24 h. Undifferentiated ESCs and EBOs cultured in medium supplemented with either 15% SR or 15% ESC-qualified FCS were fixed with 3% PFA at room temperature for 10 min. Subsequently, ESCs were permeabilized with 0.1% Triton-X 100 (Sigma Aldrich) for 5 min at room temperature, while EBs and EBOs with 0.5% Triton-X 100 (Sigma Aldrich) at room temperature for 40 min. Nonspecific antibody binding was blocked by incubation in 3% bovine serum albumin (BSA, Sigma Aldrich) in PBS at room temperature for 1 h. Next, cells were incubated at 4 °C overnight with the following primary antibodies diluted in 3% BSA in PBS: anti-BRACHYURY (10 µg/mL; R&D Systems, Minneapolis, MN, USA, AF20850), anti-GATA4 (1:100, Santa Cruz sc9053), anti-PAX6 (1:100, Santa Cruz sc81649), anti-cardiac TROPONIN T (TNNT, 1:100, Abcam ab8295), anti-β III-TUBULIN (TUJ, 1:100; Cell Signaling Technology, Danvers, MA, USA, 5568) or anti-ALPHA-FETOPROTEIN (AFP, 1:100, Santa Cruz, sc-8108). Afterward, cells were incubated with appropriate secondary antibodies conjugated with Alexa Fluor 594 (Thermo Fisher Scientific) and diluted in 3% BSA in PBS in darkness for 2 h, and then in DRAQ5 (Biostatus Limited, Shepshed, Great Britain), or Hoechst (Sigma Aldrich) diluted 1:1000 in PBS at room temperature for 10 min. Cells were analyzed using Axio Observer Z1 LSM 700 scanning confocal microscope (Zeiss, Oberkochen, Germany) with ZEN software. 

### 2.9. Western Blotting

Proteins were isolated from EBs cultured in a medium supplemented with either 15% SR or 15% ESC-qualified FCS with mirVana Paris kit (Thermo Fisher Scientific) with protease (Complete Mini, Roche) and phosphatase inhibitors (PhosSTOP, Roche). Protein concentration was measured with Pierce BCA Protein Assay Kit (Thermo Fisher Scientific). Ten µg of total protein lysate was denatured by boiling in Laemmli buffer at 100 °C for 10 min and separated using SDS-PAGE electrophoresis and transferred to polyvinylidene difluoride membrane (Bio-Rad, Hercules, CA, USA). The membranes were blocked with 5% skimmed milk in TBS at room temperature for 1 h and incubated with primary antibodies diluted in 2.5% skimmed milk in TBS at four °C overnight. Next, membranes were incubated with appropriate peroxidase-conjugated secondary antibody diluted in 5% skimmed milk in TBS at room temperature for 2 h. For non-phospho β-CATENIN and pJNK detection, membranes were blocked and incubated with antibodies in 5% BSA and 0.1% Tween^®^20 (Sigma Aldrich) in TBS according to the manufacturer’s instructions. Next, protein bands were visualized with SuperSignal™ West Dura Extended Duration Substrate (Thermo Fisher Scientific) and exposed to chemiluminescence positive film (Amersham Hyperfilm ECL, GE Healthcare, Chicago, IL, USA), which was developed with standard photographic agents (Fuji). Antibodies against the following antigens were used: non-phospho (active) β-CATENIN (ABC, diluted 1:1000, Cell Signaling Technology, 19807), phospho-SAPK/JNK (pJNK, diluted 1:1000, Cell Signaling Technology, 9251), TUBULIN (diluted 1:1000, Sigma Aldrich T5168), HSP90 (diluted 1:2000, OriGene Technologies, Rockville, MD, USA, TA500494). Optical density of bands was measured using GelDocXR+ (Bio-Rad) with ImageLab software.

### 2.10. ELISA

SHH concentration was analyzed with Mouse Sonic Hedgehog/SHH N-Terminus Quantikine ELISA Kit (R&D Systems MSHH00) according to the manufacturer’s protocol. Supernatants were collected from undifferentiated ESCs, EBs, and EBOs cultured in medium supplemented with either 15% SR or 15% ESC-qualified FCS. Additionally, both types of cultural media were also analyzed. The experiment was repeated thrice. The plate was analyzed with Gen5 Microplate Reader and Image software. 

### 2.11. Statistical Analysis

All experimental procedures were performed at least three times. Results are presented as mean ± standard deviation (SD). Data was analyzed with GraphPad Prism 8 software. The normality of data distribution was tested with the Shapiro–Wilk test. For the data that was distributed normally, the significance was calculated with a non-paired Student’s *t*-test. For the data sets that did not follow the normal distribution, the Mann—Whitney U test was applied. Data found to be statistically significant are marked with asterisks (* *p* < 0.05; ** *p* < 0.01; *** *p* < 0.001; **** *p* < 0.0001).

## 3. Results

### 3.1. ESCs Preferentially Differentiate into Ectodermal Lineage under Defined Culture Conditions

In the initial set of experiments, we compared proliferation and differentiation of mouse ESCs in EBs and EB-derived outgrowths (¬EBOs), cultured in two types of medium, i.e., (1) medium supplemented with serum (FCS, fetal calf serum) and (2) chemically defined medium supplemented with serum replacement (SR). Cells were analyzed after 2, 5, or 7 days of EB culture or after further 7 days of EBO culture.

**Figure 1:**
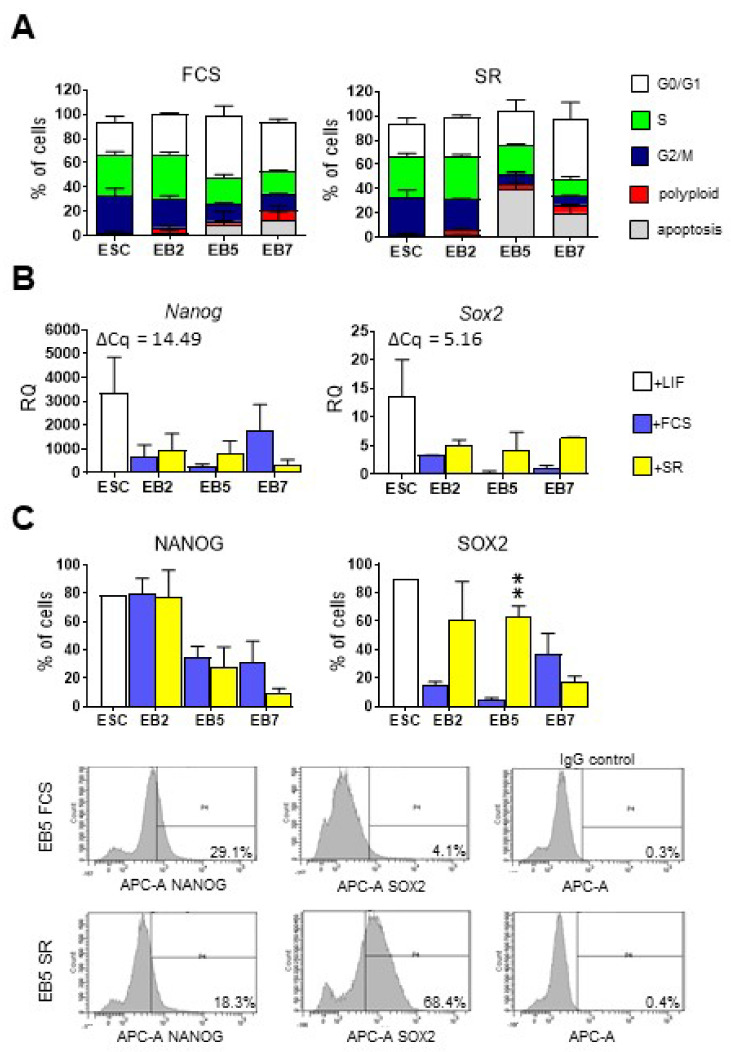
Analysis of cell cycle and pluripotency marker expression in undifferentiated ESCs and EBs cultured in medium supplemented either with FCS or SR. (**A**) The proportion of cells in different stages of cell cycle analyzed by propidium iodide staining. On the graph, the proportion of cells during each cell cycle phase is presented: G0/G1, S, G2/M as well as apoptotic and polyploid cells. (**B**) Expression of pluripotency markers: *Nanog* and *Sox2*. *β-actin* was used as a reference gene. RQ = 1 for the level of gene expression detected in a 13.5-day-old mouse embryo (reference sample). The average ΔCq for reference sample is shown on each graph. Data presented as means of three independent experiments with standard deviations. (**C**) The proportion of undifferentiated ESCs and cells obtained after disaggregation of EB2, EB5, and EB7 synthesizing NANOG or SOX2, analyzed with flow cytometry. Data presented as means of three independent experiments with standard deviations; ** *p* < 0.01. Representative histograms present proportion of EB5-derived cells synthesizing NANOG or SOX2, and cells incubated with IgG only (negative control).

ESCs were able to form EBs regardless of medium type. We found that EBs formed in SR-supplemented medium were smaller and more compacted at day 2 of the culture, but at other time-points no differences, neither in EB morphology, nor size, nor cell number were observed between cells cultured in the presence of either SR or FCS (data not shown). By performing propidium iodide staining, we assessed the proportion of cells in G0/G1, S, and G2/M phases and polyploid and apoptotic ones in undifferentiated ESCs and EBs cultured in a medium supplemented either with SR or FCS. Regardless of medium type, the proportion of cells in the S phase was the highest in undifferentiated ESCs (33.85% ± 2.3%) while in EBs decreased gradually: in EB5 and EB7 the majority of cells was in G0/G1 phase. We noticed a higher proportion of apoptotic cells in EB5 cultured in the presence of SR than in EB5 cultured in medium supplemented with FCS, but the observed differences were statistically insignificant. Moreover, we counted cells after Trypan blue staining and found no significant differences in the number of lives as well as dead cells between both culture conditions (data not shown). Therefore, the impact of SR on EB size as well as apoptosis was noticeable only at one time-point, transient and not significant. In general, no significant differences between EBs cultured in medium supplemented either with SR or FCS were found (Figure 1A). 

Next, we analyzed the expression levels of *Nanog* and *Sox2*, major pluripotency regulators [35,36]. Level of both transcripts was high in undifferentiated ESCs and decreased in EBs: no statistically significant differences were found between cells cultured in the presence of either SR or FCS (Figure 1B). Flow cytometry analysis confirmed that the proportion of cells synthesizing NANOG was high in undifferentiated ESCs and decreased during EB differentiation. Again, no significant differences between cells cultured in different media were found (Figure 1C). In the case of SOX2 proportion of cells synthesizing this protein was significantly higher in EBs cultured in a medium supplemented with SR at day 5 of the culture (Figure 1C).

**Figure 2:**
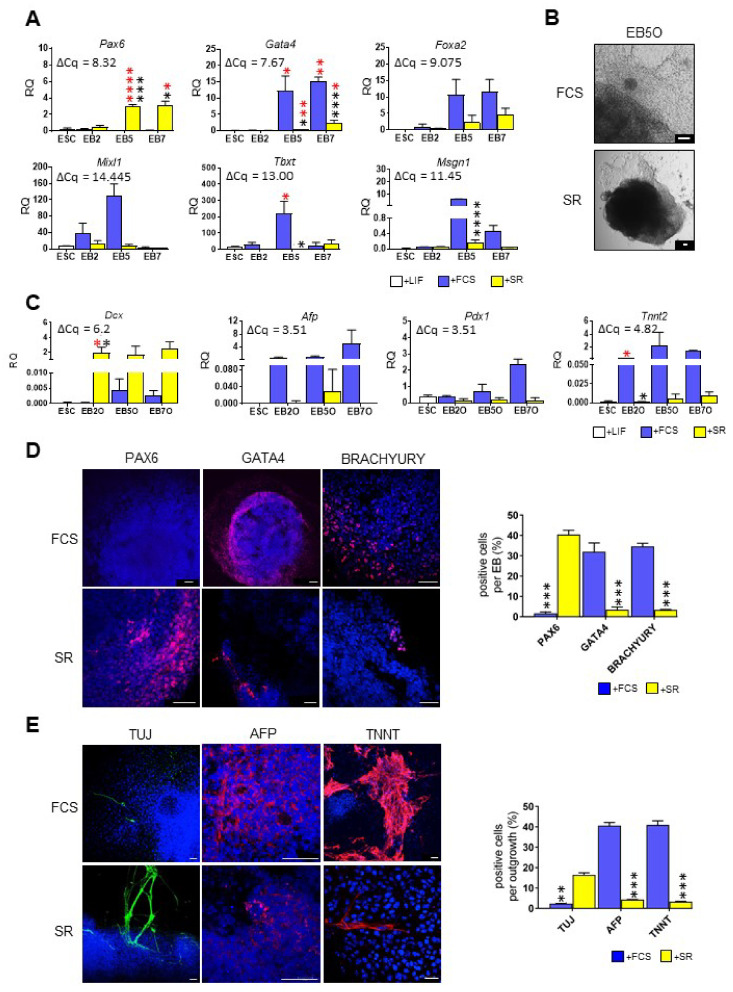
Analysis of differentiation marker expression in undifferentiated ESCs, EBs and EBOs cultured in medium supplemented either with FCS or SR. (**A**) Expression of germ layer markers: *Pax6*, *Gata4*, *Foxa2*, *Mixl1*, *Tbxt*, *Msgn1*. (**B**) Morphology of EBOs derived from EB5. Scale bar: 100 µm. (**C**) Expression of selected cell type markers: *Dcx*, *Afp*, *Pdx1*, *Tnnt2*. (**D**) Representative images of PAX6, GATA4, and BRACHYURY immunostaining in EB5. Scale bar: 50 µm. Analyzed proteins are shown in red; DNA in blue. The graph shows the mean proportion of cells expressing indicated proteins in EBs. (**E**) Representative images of TUJ, AFP, and TNNT immunostaining in EB5O. Analyzed proteins are shown in green (TUJ) or red (AFP and TNNT); DNA in blue. Scale bar: 50 µm. The graph shows the mean proportion of cells expressing indicated proteins in outgrowth. For the qPCR analyses (**A**,**C**) *β-actin* served as a reference gene. RQ = 1 for the expression level detected in a 13.5-day-old mouse embryo. The average ΔCq for reference sample is shown on each graph. Data presented as means of three independent experiments with standard deviations; * *p* < 0.05; ** *p* < 0.01; *** *p* < 0.001; **** *p* < 0.0001. Red color indicates differences between undifferentiated ESCs and EBs or EBOs; black color indicates differences between cells cultured in medium supplemented either with FCS or SR.

Next, we focused on the initial stages of ESC differentiation, i.e., the formation of germ layers. The expression of *Pax6*—ectodermal marker [37], *Gata4* and *Foxa2*—endodermal markers [38,39], as well as *Tbxt* (encoding BRACHYURY protein), *Mixl1* and *Msgn1*—mesodermal markers [40,41,42] was analyzed in undifferentiated ESCs and EBs. Expression of none of these markers was detected in undifferentiated ESCs. Expression of germ layer markers in EBs cultured in the presence of either SR or FCS differed significantly. EBs cultured in the chemically defined medium were characterized by significantly higher levels of *Pax6* expression while endo- and mesodermal markers (*Gata4*, *Tbxt*, *Msgn1*) were expressed at higher levels in EBs cultured in a medium supplemented with FCS (Figure 2A). We found the same profile of expression while analyzing another mouse ESC cell line, D3 line (data not shown); thus, the observed effect did not depend on intrinsic cell line properties.

To verify whether the type of culture medium affects later stages of ESC differentiation we examined EBOs cultured in the presence of either SR or FCS for 7 days. EBOs cultured in the presence of serum were evidently bigger and more spread than EBOs cultured in the presence of SR, which were smaller and less dispersed (Figure 2B). We analyzed the expression of selected markers of specialized cells, i.e., DOUBLECORTIN, *Dcx*, marker of migrating neurons [43], *Afp*, *Pdx1,* and *TnnT2*, markers of pancreatic β cells, hepatocytes, and cardiomyocytes, respectively [44,45,46]. We found that level of *Dcx* was notably higher in EBOs cultured in a medium supplemented with SR, while expression of *Pdx1*, *Afp*, and *TnnT2* was higher in EBOs cultured in the presence of serum (Figure 2C). 

Significant differences between EBs and EBOs cultured in different media were also confirmed by immunostaining. EBs cultured in the medium supplemented with FCS were characterized by the lack of PAX6-synthesizing cells and the presence of many GATA4- or BRACHYURY-positive cells (Figure 2D). Contrary, in EBs cultured in a medium supplemented with SR, numerous PAX6-positive cells were observed while GATA4- and BRACHYURY-producing cells were scarce (Figure 2D). Then, we determined the presence of TUJ, AFP, and TNNT proteins in EBOs cultured in the presence of either SR or FCS. TUJ is a cytoskeletal protein characteristic for neurons originating from ectoderm [47]. In EBOs cultured in the presence of serum, no TUJ-positive cells were detected, while AFP and TNNT-producing cells (originating from endo- and mesoderm, respectively) were abundant (Figure 2E). In EBOs cultured in a medium supplemented with SR, cells characterized by the presence of AFP and TNNT proteins were virtually absent, while TUJ-positive cells were easily noticeable (Figure 2E).

Presented results clearly demonstrate that ESCs cultured in media selected by us underwent differentiation but in significantly different manner. Endo- and mesodermal conversion of cells cultured in the chemically defined medium was limited in comparison to cells cultured in serum-supplemented medium. In the subsequent set of experiments, we verified whether differentiation of ESCs cultured under defined conditions is influenced by important regulators, i.e., WNT and SHH proteins. 

### 3.2. Wnt Expression and Activity Significantly Differ in Mouse ESCs Cultured in the Presence of SR or FCS

**Figure 3:**
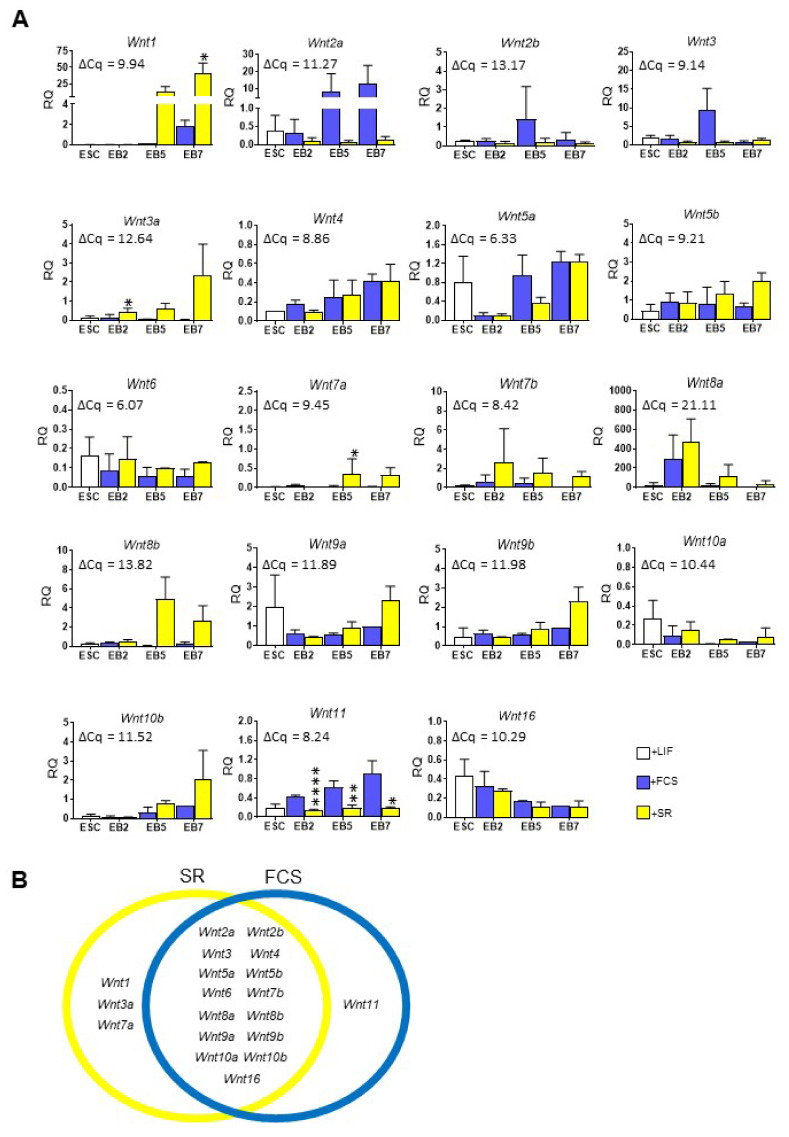
*Wnt* expression in undifferentiated and differentiating ESCs cultured in medium supplemented either with FCS or SR. (**A**) qPCR analysis. *β-actin* was used as a reference gene. RQ=1 for the level of gene expression detected in a 13.5-day-old mouse embryo. The average ΔCq for reference sample is shown on each graph. Data presented as means of three independent experiments with standard deviations; * *p* < 0.05; ** *p* < 0.01; **** *p* < 0.0001. (**B**) Graphical summary of *Wnt* expression.

We examined the expression of all 19 *Wnt* genes in both undifferentiated ESCs and cells differentiating in EBs (Figure 3A). We did not find any *Wnt* factor that was exclusively expressed in undifferentiated ESCs. Expression of almost all analyzed *Wnts* increased steadily in EBs; however, there were substantial differences between cells cultured in different media. Based on the results of statistical analysis we divided all 19 analyzed *Wnts* into three groups (Figure 3B): (1) the one that was expressed at significantly higher levels in EBs cultured in medium supplemented with FCS (*Wnt11*), (2) the ones expressed at significantly higher level in cells in SR-supplemented medium (*Wnt1*, *Wnt3a*, *Wnt7a*), and (3) the ones that were expressed in cells under both culture conditions (*Wnt2a*, *Wnt3*, *Wnt2b*, *Wnt4*, *Wnt5a*, *Wnt5b*, *Wnt6*, *Wnt7b*, *Wnt8a*, *Wnt8b*, *Wnt9a*, *Wnt9b*, *Wnt10a*, *Wnt10b*, *Wnt16*). In most cases, the level of Wnts expressed in undifferentiated and differentiating ESCs can be assessed as moderate when compared to the average value of ΔCq for reference sample (13.5-day-old mouse embryo).

**Figure 4:**
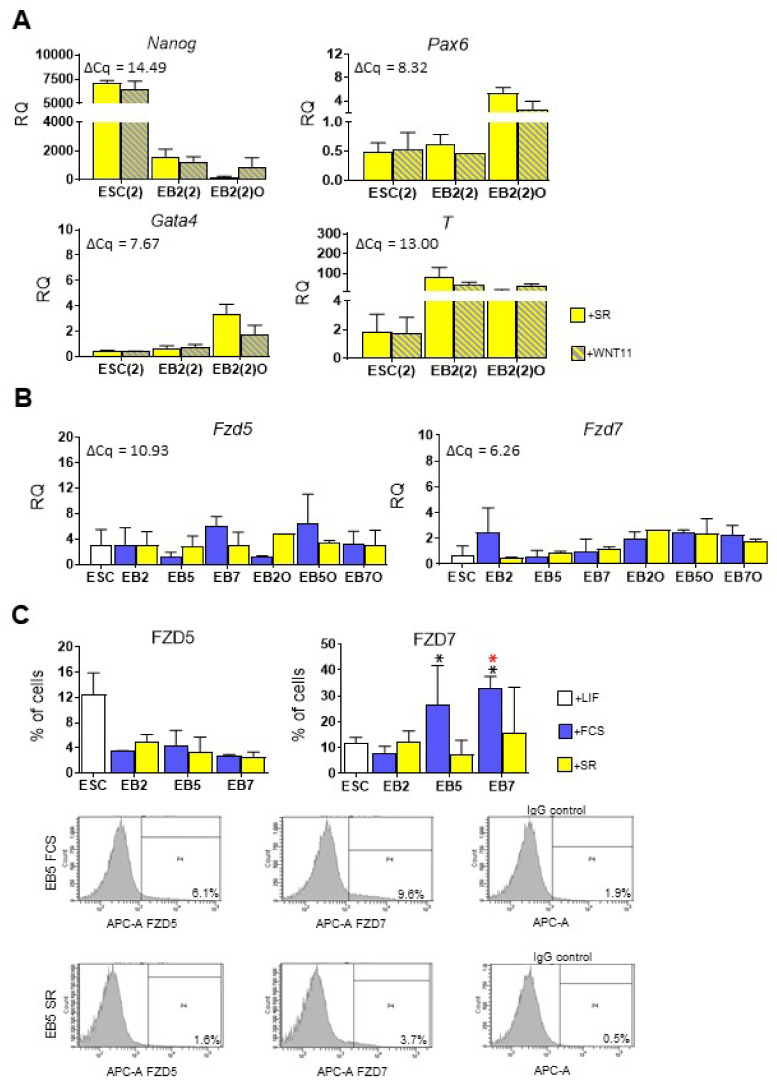
WNT11 treatment and analysis of WNT11 receptors in undifferentiated and differentiating ESCs. (**A**) Expression of *Nanog*, *Pax6*, *Gata4* and *T* in WNT11-treated and control ESCs, EBs and EBOs cultured in medium supplemented with SR. Cells were collected after 48h of WNT11 treatment. (**B**) Expression of WNT11 receptors: *Fzd5* and *Fzd7*. (**C**) FACS analysis: proportion of undifferentiated ESCs and cells obtained after disaggregation of EB2, EB5, and EB7 synthesizing FZD5 or FZD7. Data presented as means of three independent experiments with standard deviations. Representative histograms present proportion of EB5-derived cells synthesizing FZD5 or FZD7 or cells incubated with IgG only (negative control). Cells were cultured in medium supplemented either with SR or FCS. For qPCR analyzes (**A**,**B**) *β-actin* was used as a reference gene. RQ = 1 for the level of gene expression detected in a 13.5-day-old mouse embryo. The average ΔCq for reference sample is shown on each graph. Data are presented as means of three independent experiments with standard deviations. * *p* < 0.05; Red color indicates differences between undifferentiated ESCs and EBs or EBOs; black color indicates differences between cells cultured in medium supplemented either with FCS or SR.

Since high expression of endo- and mesodermal markers in ESCs differentiating in medium supplemented with FCS correlated with higher expression level of *Wnt11* than in cells cultured in the presence of SR, in the following set of experiments, we verified whether addition of WNT11, known as engaged in early muscle fibre organization [48], to chemically defined culture medium could influence on ESC mesodermal differentiation, which was limited under such culture conditions. To this end, we analyzed the expression of pluripotency and germ layer markers in cells cultured in medium supplemented with SR and exogenous WNT11 for two days: ESCs cultured in monolayer (referred to as ESC(2)), EB2 (referred to as EB2(2)), and EB2-derived outgrowths (referred to as EB2(2)O). We did not observe any significant effect of WNT11 treatment on differentiating ESC (Figure 4A). This could be explained by the fact that only small populations of undifferentiated and differentiating ESCs cultured in the presence of SR synthesize receptors for WNT11: FRIZZLED-5 (FZD5 [49]) and FRIZZLED-7 (FZD7 [50]) (Figure 4B,C). Therefore, only a small proportion of treated cells could respond to recombinant WNT11 added to the culture medium. In case of the ESCs cultured in the presence of FCS we found a higher number of cells synthesizing FZD7 at day 5 and 7 of EB culture (Figure 4C). Thus, a higher level of *Wnt11* expression in EB cultured in the presence of FCS than in cells cultured in the SR medium was accompanied by higher number of cells synthesizing its receptor—FZD7, at days 5 and 7 of the culture. As a result, there were more cells that could respond to WNT11 action in EBs cultured in the presence of FCS.

**Figure 5:**
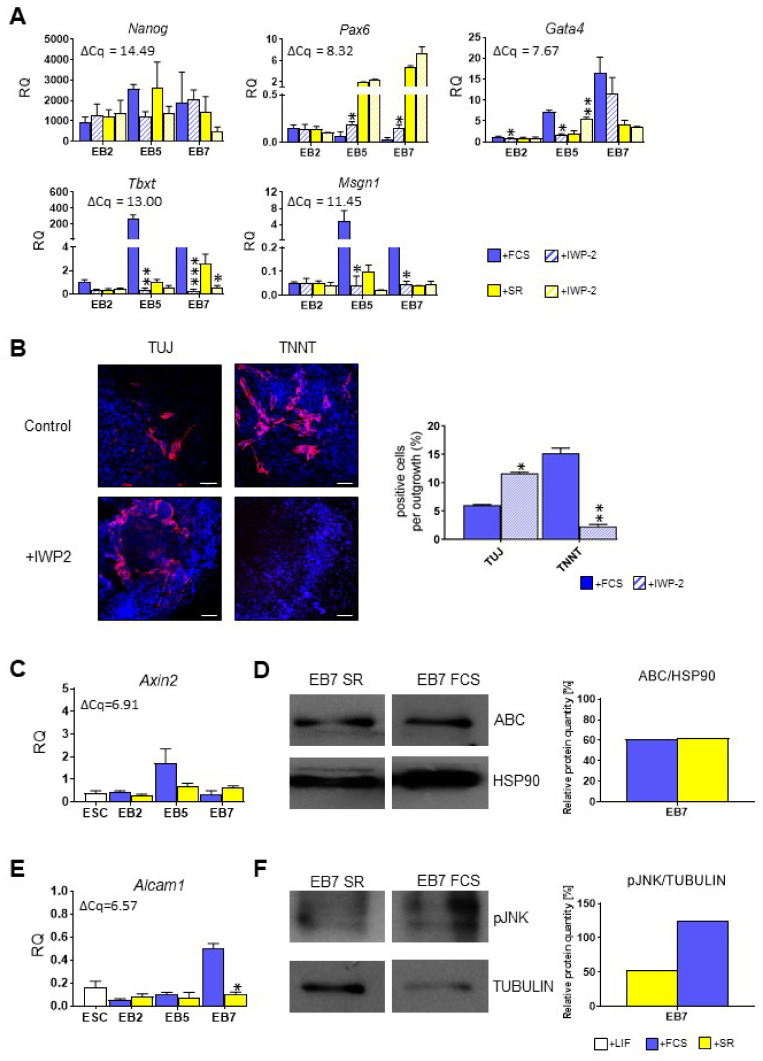
Analysis of WNT secretion inhibition and WNT pathway activity in mouse ESCs cultured in medium supplemented either with FCS or SR. (**A**) Expression of *Nanog*, *Pax6*, *Gata4*, *Tbxt*, and *Msgn1* in control and IWP-2-treated EBs. (**B**) Representative images of TUJ and TNNT immunodetection in outgrowths derived from control and IWP-2 treated EB5 cultured in the presence of FCS. Analyzed proteins are shown in red; DNA in blue. Scale bar: 50 µm. The graph shows the mean proportion of cells expressing indicated proteins in outgrowth. (**C**) Expression of *Axin2* in undifferentiated ESCs and EBs. (**D**) Level of active (non-phosphorylated β-CATENIN; ABC) and HSP90 in EBs. HSP90 was used as a loading control. Relative protein quantity ABC/HSP90 is shown on the graph. (**E**) Expression of *Alcam1* in undifferentiated ESCs and EBs. (**F**) Level of phosphorylated JNK (pJNK) and TUBULIN in EBs. TUBULIN was used as a loading control. Relative protein quantity pJNK/TUBULIN is shown in the graph. (**A**,**C**,**E**) For qPCR analyses *β-actin* was used as a reference gene. RQ = 1 for the expression level detected in a 13.5-day-old mouse embryo. The average ΔCq for reference sample is shown on each graph. Data presented as means of three independent experiments with standard deviations; * *p* < 0.05; ** *p* < 0.01; *** *p* < 0.001.

In the subsequent experiments, we verified whether WNT factors are indeed involved in ESC mesodermal differentiation or whether their presence is dispensable for this process. To this aim, we blocked endogenous WNT signaling in ESCs differentiating in EBs by using IWP-2 inhibitor. IWP-2, by specifically binding the porcupine enzyme, which palmitoylates WNT proteins, blocks their secretion and, therefore, activity [51]. Next, we studied the expression of selected pluripotency and differentiation markers in control EBs and EBs cultured in the presence of IWP-2 in media supplemented either with FCS or SR for 2, 5 or 7 days. We found that IWP-2 treatment had no significant impact on the expression of *Nanog*, pluripotency marker (Figure 5A). Still, it increased *Pax6* expression in EB5 and EB7 cultured in medium supplemented with FCS (Figure 5A). The level of *Pax6* expression in such cells was still much lower in comparison to cells cultured in the presence of SR; however, not negligible. WNT inhibition had an opposite effect on *Gata4* expression in both groups of EB5: it significantly decreased *Gata4* expression in EB5 cultured in medium supplemented with FCS while elevated its level in cells cultured in chemically defined medium (Figure 5A). It should be noted that level of *Gata4* expression in EB5 as well as in EB7 was quite high, as the average ΔCq for reference sample was 7.67. Most importantly, blocking WNT secretion led to the inhibition of ESC mesodermal differentiation: neither *Tbxt* nor *Msgn1* expression was detected in IWP-2-treated EB5 and EB7 (Figure 5A).

To verify whether the abovementioned changes in gene expression observed in IWP-2 treated EBs influenced their fate, we performed immunocytochemical analysis and found that cells cultured in FCS and treated with IWP-2 formed more TUJ-positive cells (originating from ectoderm) and few or any TNNT-positive cells (originating from mesoderm) in comparison to untreated cells (Figure 5B). These results confirmed the pivotal role of active WNT signaling in inducing mesodermal differentiation of mouse PSCs.

To elucidate which type of WNT signaling pathways are the core regulators during ESC mesodermal differentiation, we determined the activity of both canonical WNT and noncanonical WNT/JNK pathways in ESCs differentiating in EBs. We did not observe any significant differences in the expression of *Axin2*, a known target gene in the canonical WNT pathway [52], between cells differentiating in the presence of either SR or FCS at any studied time point (Figure 5C). In addition, no differences in the level of active, non-phosphorylated form of β-CATENIN were observed in EBs cultured under both conditions (Figure 5D). Contrary to this, the expression of *Alcam1*, a known target gene of WNT/JNK pathway [53], was significantly higher in EB7 cultured in the presence of serum (Figure 5E). Moreover, pJNK isoforms were more abundant in such cells (Figure 5F). These results suggested that more efficient ESC differentiation into mesodermal lineage correlates with higher activity of WNT/JNK. 

### 3.3. Both Canonical and Noncanonical WNT Signaling Pathway Are Crucial for ESC Differentiation into Mesoderm

To further study the role of both canonical and noncanonical WNT pathways, we determined how their inhibition influenced ESC differentiation in media selected by us. To inhibit canonical and noncanonical WNT/JNK pathways we used small-molecule inhibitors: IWR-1 and SP600125, respectively. IWR-1 stabilizes protein complex phosphorylating β-CATENIN, which leads to its degradation in the proteasome [51], while SP600125 blocks JNK phosphorylation and activation [54]. To verify the influence of these inhibitors (as well as IWP-2 previously described), we assessed the level of *Alcam1* and *Axin2* in treated cells as well as their nmber after Trypan blue staining (Supplementary Figure S2A,B).

**Figure 6:**
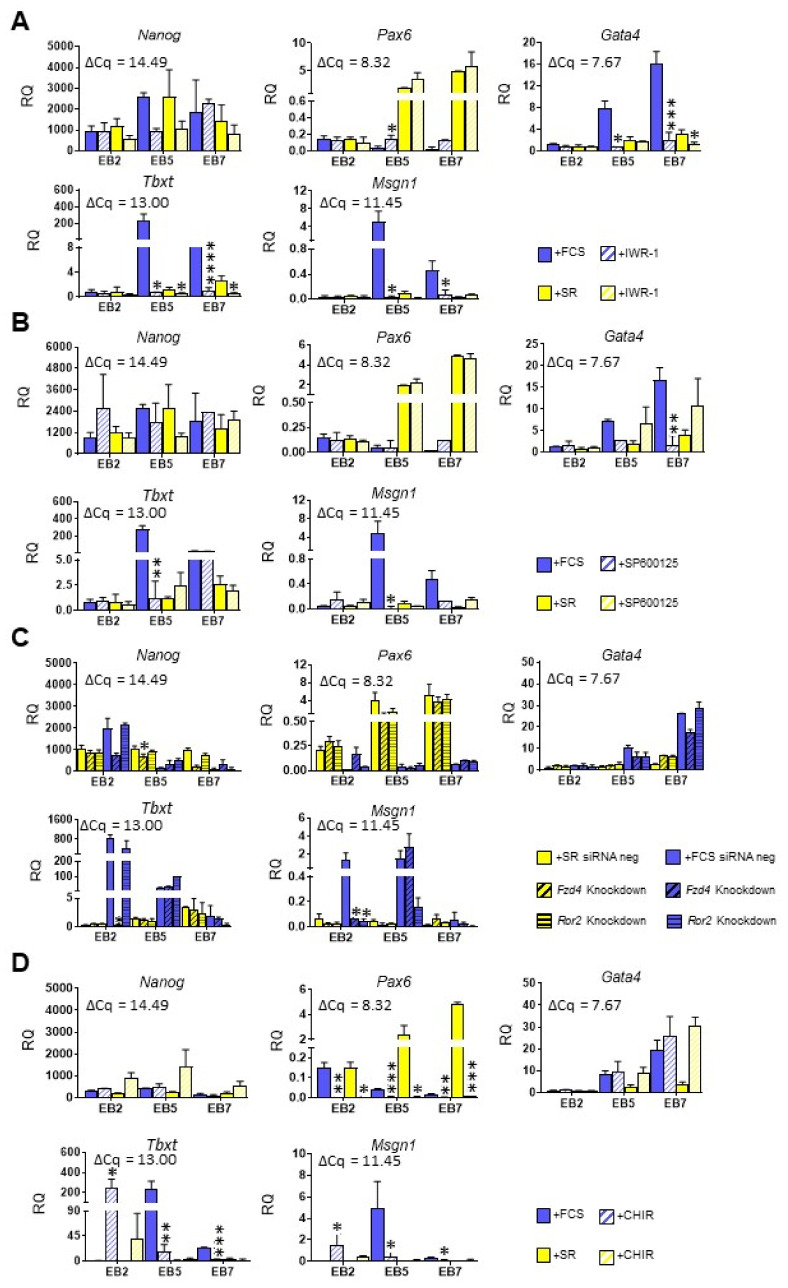
Analysis of ESC differentiation in medium supplemented either with FCS or SR after modification of either canonical or noncanonical WNT pathway. (**A**) Expression of germ layer markers in control or IWR-1-treated EBs. (**B**) Expression of germ layer markers in control or SP600125-treated EBs. (**C**) Expression of germ layer markers in control or siRNA-transfected EBs. (**D**) Expression of germ layer markers in control or CHIR-treated EBs. *β-actin* was used as a reference gene. RQ = 1 for the expression level detected in a 13.5-day-old mouse embryo. The average ΔCq for reference sample is shown on each graph. Data presented as means of three independent experiments with standard deviations; * *p* < 0.05; ** *p* < 0.01; *** *p* < 0.001; **** *p* < 0.0001.

To determine the effect of WNT signaling inhibition on ESC differentiation we studied the expression of selected pluripotency and differentiation markers in control EBs and EBs cultured in the presence of indicated inhibitors for 2, 5, or 7 days. No significant changes in *Nanog* expression were observed after canonical pathway inhibition regardless of culture medium. In cells cultured in FCS-supplemented medium, a significant increase in *Pax6* expression was observed in treated EB5, although, it still remained much lower than in cells cultured in the presence of SR (Figure 6A). WNT canonical pathway inhibition resulted in a significant decrease in expression of endo- and mesodermal markers in EB5 and EB7 cultured in both types of media; however, the observed changes were much lower in cells cultured in the presence of SR due to the initial low expression level of these markers (Figure 6A).

In EBs, in which the noncanonical WNT pathway was blocked, we did not observe any significant differences in *Nanog* and *Pax6* expression between cells cultured in different media (Figure 6B). Expression of *Gata4* (endodermal), *Tbxt* and *Msgn1* (mesodermal markers) was significantly reduced in EB5 and EB7 cultured in the medium supplemented with FCS (Figure 6B). These observations were partially confirmed by siRNA-mediated knockdown of *Fzd4* or *Ror2* (Supplementary Figure S2C) in ESC differentiating in EBs. FZD4 and ROR2 bind the same Wnt, i.e., Wnt5a, but activate canonical or noncanonical WNT pathway, respectively [55]. *Ror2* knockdown significantly decreased *Msgn1* expression in EB2 cultured in the presence of FCS while *Fzd4* knockdown reduced expression of both mesodermal markers, *Tbxt* and *Msgn1* (Figure 6C).

Next, we determined the effect of WNT signaling activation on ESC fate. ESCs differentiating in EBs were cultured in the presence of CHIR99021, canonical WNT pathway activator, for 2, 5, or 7 days. CHIR99021 is a GSK3β inhibitor that leads to the destabilization of β-CATENIN phosphorylating complex [56]. We demonstrated that such treatment did not affect the expression of *Nanog*, but it lead to the significant decrease in the expression of *Pax6* in EBs at all studied time points, regardless of medium type. No significant differences in *Gata4* expression were observed, while CHIR treatment markedly increased expression of *Tbxt* and *Msgn1*, i.e., mesodermal markers in EB2 cultured in the presence of FCS or SR, respectively (Figure 6D). However, it is important to emphasize that induction of mesodermal markers was transient, as treatment with CHIR for 5 or 7 days led to the opposite effect in EBs cultured in media supplemented with FCS, i.e., it significantly decreased expression of *Tbxt* and *Msgn1* in these cells (Figure 6D). Meanwhile, activating noncanonical WNT signaling with anisomycin, i.e., JNK activator [57], led to the apoptosis of differentiating ESCs (data not shown). Therefore, we were not able to determine the effect of the direct activation of the noncanonical WNT pathway in ESCs differentiating in EBs.

### 3.4. Higher SHH Expression and Activity Corresponds with Preferential Ectodermal Differentiation of Mouse ESCs

**Figure 7:**
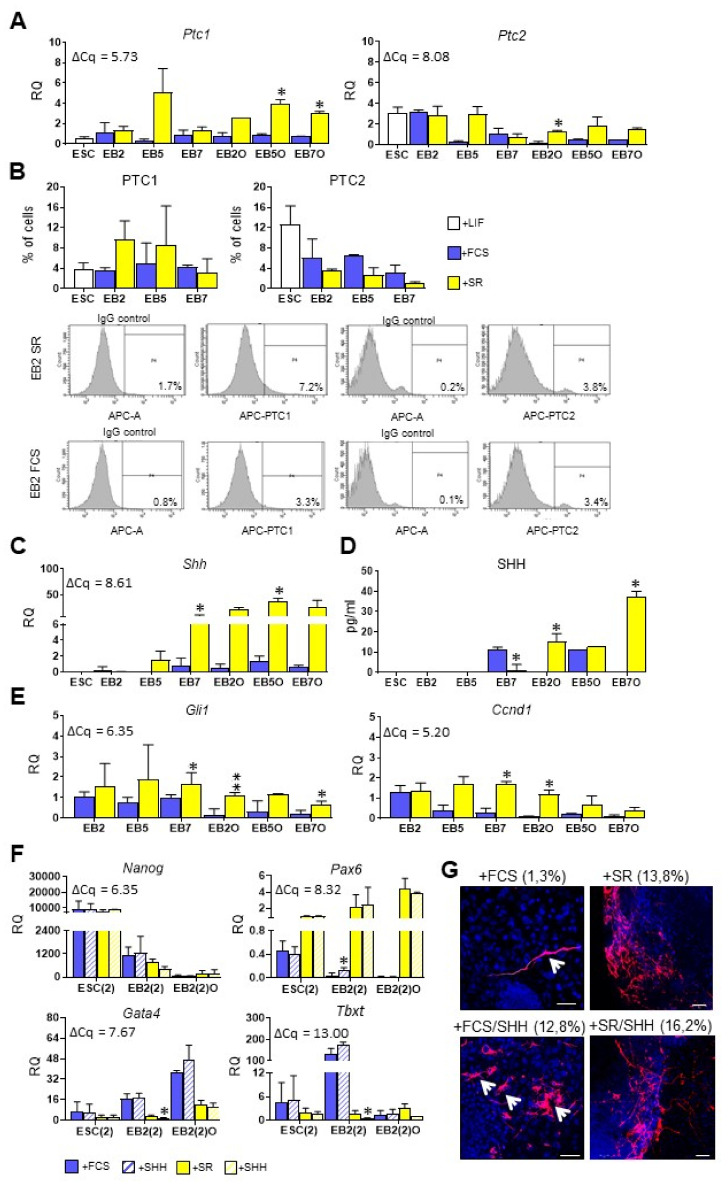
Analysis of SHH, its receptors and SHH pathway activity in undifferentiated and differentiating mouse ESCs cultured in medium supplemented either with FCS or SR. (**A**) Expression of SHH receptors: *Ptc1* and *Ptc2* in ESCs, EBs and EBOs. (**B**) The proportion of undifferentiated ESCs and cells obtained after disaggregation of EB2, EB5, and EB7 synthesizing either PTC1 or PTC2, analyzed with flow cytometry. Representative histograms present the percentage of EB2-derived cells synthesizing PTC1 or PTC2 or cells incubated with IgG only (negative control). (**C**) Expression of *Shh* in ESCs, EBs and EBOs. (**D**) SHH concentration in the medium collected either from undifferentiated ESCs or EBs or EBOs. (**E**) Expression of *Gli1* and *Ccnd1*. (**F**) Expression of *Nanog*, *Pax6*, *Gata4*, *Tbxt* in ESCs, EBs and EBOs treated with recombinant SHH for 48h and control ones. (**G**) Representative images of immunocytochemistry analysis of TUJ in EB2(2)O treated with SHH and control ones, cultured in medium supplemented with either FCS or SR. TUJ protein in red; DNA in blue. Scale: 50 μm. Numbers in brackets indicate the average proportion of TUJ positive cells in outgrowths. For qPCR analysis (**A**,**C**,**E**,**F**) *β-actin* was used as a reference gene. RQ = 1 for the level of gene expression detected in a 13.5-day-old mouse embryo. The average ΔCq for reference sample is shown on each graph. Data presented as means of three independent experiments with standard deviations; * *p* < 0.05, ** *p* < 0.01.

As mentioned above, SHH is a known regulator of mesodermal/myogenic differentiation, acting together with WNT proteins [58]. For this reason, we studied the expression of *Shh* and its receptors: *Ptc1* and *Ptc2* in undifferentiated ESCs, EBs cultured for 2, 5 or 7 days, and EB-derived EBOs. Importantly, expression of *Ptc1* was high in undifferentiated ESCs and remained constant in EBs and EBOs cultured in medium supplemented with FCS, while it increased markedly in EB5O and EB7O cultured in medium with SR (Figure 7A). *Ptc2* expression was also high in undifferentiated ESCs and remained at the same level in differentiating cells cultured in medium supplemented either with FCS or SR (Figure 7A). Subsequent flow cytometry analysis confirmed that a subpopulation of undifferentiated ESCs as well as cells building EBs synthesizes PTC receptors (Figure 7B). No significant differences in the proportion of cells synthesizing either PTC1 or PTC2 were observed between both undifferentiated and differentiating ESCs as well as between cells cultured in media supplemented with either FCS or SR. Expression of *Shh* was undetected in undifferentiated ESCs and remained low in ESCs differentiating in EBs cultured in medium with FCS, while it increased significantly in EBs and EBOs cultured in the presence of SR (Figure 7C). These results were confirmed by the ELISA analysis: SHH protein was undetectable in undifferentiated ESCs-conditioned medium while its concentration increased in medium collected from EB2O and EB7O cultured in the presence of SR while remained low in the medium conditioned by their counterparts, cultured in the presence of FCS (Figure 7D). To confirm the activity of the SHH signaling pathway in differentiating ESCs we also studied the expression of two genes activated by this pathway: *Gli1* [28] and *Ccnd1* [59]. Importantly, the expression of both genes was significantly higher in EB7 and EBOs cultured in a medium supplemented with SR (Figure 7E). Therefore, our results suggested that SHH is highly expressed and active in EBs and EBOs cultured in chemically defined medium, which is characterized by a high level of ectodermal marker expression.

In the final set of experiments, we studied the influence of 48-h-long exogenous SHH protein treatment on differentiation of ESCs: cultured in monolayer (ESC(2)), differentiating in EB2 (EB2(2)) and EB-derived outgrowths (referred to as EB2(2)O). We showed that SHH did not affect the expression of *Nanog*, a pluripotency marker, but an elevated expression of *Pax6* in SHH-treated EB2(2) cultured in medium with FCS (Figure 7F). However, the level of *Pax6* expression in such cells was still much lower than in cells cultured in the presence of SR; we detected numerous TUJ-synthesizing neurons in outgrowths derived from cells cultured in the presence of FCS and treated with SHH while only a few such cells were found in outgrowths derived from untreated cells cultured in the presence of FCS (Figure 7G). Abundant TUJ positive cells were also present in the outgrowths derived from cells cultured in the presence of SR, both untreated as well as treated with exogenous SHH (Figure 7G). Exogenous SHH decreased expression of both *Gata4* and *Tbxt* in EB2(2) cultured in medium with SR, although it should be noted that the initial expression level of these markers was low in these cells (Figure 7F). Therefore, under the culture conditions used in the current study SHH was found to be an inducer of ESC ectodermal rather than mesodermal differentiation.

## 4. Discussion

For many years, protocols for efficient PSC differentiation into desired cell populations have been studied and optimized. Despite recent progress, no robust and clinically safe protocols have been proposed for derivation of many cell types, including skeletal muscle myoblasts. Many of the previously mentioned hurdles still remain, among them one of the most important is PSC differentiation under fully-defined conditions, without animal-derived reagents. Many of the PSC studies are still conducted with the use of serum-supplemented media. This limits the application of cells cultured under such conditions in potential therapies. It was previously demonstrated that PSCs propagated in the presence of animal-derived reagents incorporate xenoantigens, which might lead to their rejection after subsequent transplantation [60]. Additionally, different batches of serum may vary in concentration of growth factors, such as TGFβ, which might impact PSC differentiation and contribute to the variability between different protocols [61]. Therefore, in our work, we focused on the differentiation of ESCs cultured in chemically defined media. However, we used mouse cells that were cultured in animal-free conditions, i.e., without animal-derived reagents. To date, many chemically defined culture media have been proposed for PSC culture, such as SR-conditioned medium, TeSR, E8, or APEL media [15,62,63,64]. Such media were shown to promote PSC proliferation and pluripotency and, among them, SR proved to be the most cost-effective [65]. For this reason, we decided to focus on this reagent. 

It was previously reported that SR-supplemented medium can diminish PSC ability to form EBs [66]. However, mouse ESCs cultured by us in such a medium were able to form EBs. Additionally, we showed that ESC underwent spontaneous differentiation in EBs cultured in chemically defined medium, as was evidenced by the decrease in the proportion of cells in the S phase and increase in the proportion of cells in G0/G1 cells, similarly to the cells cultured in medium with FCS. However, we observed substantial differences between ESCs differentiating in media selected by us. Cells building EBs cultured in the presence of serum differentiated more efficiently into endo- and mesoderm. Their counterparts cultured under chemically defined conditions were characterized by higher expression of *Pax6*, a neuroectodermal marker.

Additionally, in EBOs cultured in the chemically defined medium, we observed numerous TUJ-positive cells. These findings correspond with the work by Watanabe and colleagues, who attended efficient conversion of ESC into telencephalic precursors in cell aggregates cultured in an SR-supplemented medium [67]. In our studies, to potentially enhance mesodermal differentiation of ESCs, which is a prerequisite ultimately enabling myogenic differentiation, we used factors involved in the formation of these cell lineages during development. We focused on WNTs and SHH, which were previously shown to act together as regulators of mesoderm/skeletal muscle formation [58,68]. First, we determined the endogenous expression levels of all *Wnt* factors in undifferentiated ESCs and EBs cultured in media selected by us. The vast body of evidence suggests that canonical WNT signaling plays an important role in maintaining PSC pluripotency. At the same time, other results point to its importance in inducing mesoderm formation (summarized in [69]). 

In our study, expression of several *Wnts* was detected in undifferentiated ESCs but at a low or moderate level. Expression of most of these factors increased markedly in EBs. In addition, the activity of canonical WNT signaling, as demonstrated by *Axin2* expression, was moderate in undifferentiated cells. These discrepancies can be explained by the findings of Kim and co-workers, which suggest that retention of β-CATENIN in the cytoplasm ensures pluripotency maintenance, while its translocation to the nucleus triggers PSC differentiation [70]. It can also be hypothesized that other signaling pathways, acting together with canonical WNT, play a role in determining cell fate. In EBs we did not observe any significant differences in expression of *Wnts*, which were previously reported to activate canonical WNT signaling (e.g., *Wnt1*, *Wnt3a,* and *Wnt8a*) [71], while the *Wnt11*, “noncanonical” one, was expressed at higher level in EBs cultured in medium supplemented with serum. It correlated with higher level of endo- and mesodermal markers in such cells.

Additionally, the noncanonical WNT pathway activity was significantly higher in cells differentiating in the presence of serum and blocking this pathway by SP600125 inhibitor decreased the expression of mesodermal markers, i.a., *Tbxt* and *Msgn1*. The study published by Mazzotta and colleagues showed that the WNT/JNK pathway is activated during hESC mesodermal differentiation, yet blocking it did not prove to have a big impact, suggesting that this signaling pathway may play a redundant role [72]. However, in our study, inhibition of WNT/JNK pathway had a dramatic effect on mesodermal differentiation of mouse ESCs: expression of both *Tbxt* and *Msgn1* was not detectable. These discrepancies may reflect differences between animal and human cells, including their response to different factors, as well as the importance of signaling pathways regulating their fate. 

While studying *Shh* expression, we found it significantly higher in EBs and EBOs cultured in the medium supplemented with SR. Similarly, expression of *Ccnd1* and *Gli1*, markers of SHH pathway activity, was significantly higher in cells cultured under such conditions. It was demonstrated previously that SHH, acting together with retinoic acid, promotes ESC differentiation into motor neurons [29]. Therefore, under chemically defined culture conditions, ESCs differentiate into ectoderm, a phenomenon that can be explained by the spontaneous activation of SHH signaling pathway and its high level in these cells. Consequently, in EBs cultured in medium supplemented with serum, in which SHH activity was significantly lower, differentiation into ectoderm was reduced. This observation is further supported by the phenotype of ESCs, which lack SMOOTHENED- SHH activator: such cells demonstrated impaired differentiation into neuroectoderm [73]. On the other hand, cells cultured in the presence of FCS and treated by us with exogenous SHH generated numerous TUJ-positive cells, which were scarcely formed by control untreated cells cultured in FCS. Thus, ectodermal differentiation of cells cultured in FCS and treated with SHH was enhanced. 

To summarize, our studies demonstrated substantial differences between ESC differentiated in two types of culture media. We showed that observed differences can be attributed to the activity of signaling pathways, such as canonical and noncanonical WNTs and SHH, which are activated differently in cells cultured in media selected by us. These endogenous differences can influence the differentiation potential of PSCs and, importantly, their response to the applied differentiation inducers, tested during the designing of protocols for efficient PSC differentiation.

## Data Availability

Not applicable.

## Supplementary Materials

The following are available online at https://www.mdpi.com/article/10.3390/cells10102743/s1, Figure S1: Outline of experiments, Figure S2: Influence of Wnt inhibitor or siRNA treatment on ESCs, Table S1: Taqman assays used in qPCR analyzes.
