# Supplementary material for: Comparison of Differentiation Pattern and WNT/SHH Signaling in Pluripotent Stem Cells Cultured under Different Conditions
Source: Cells. 2021 Oct 14;10(10):2743. doi: 10.3390/cells10102743 (PMC8534321; doi:10.3390/cells10102743)
Supplement: Supplementary file 1 [file cells-10-02743-s001.zip › cells-1324690-supplementary.pdf]

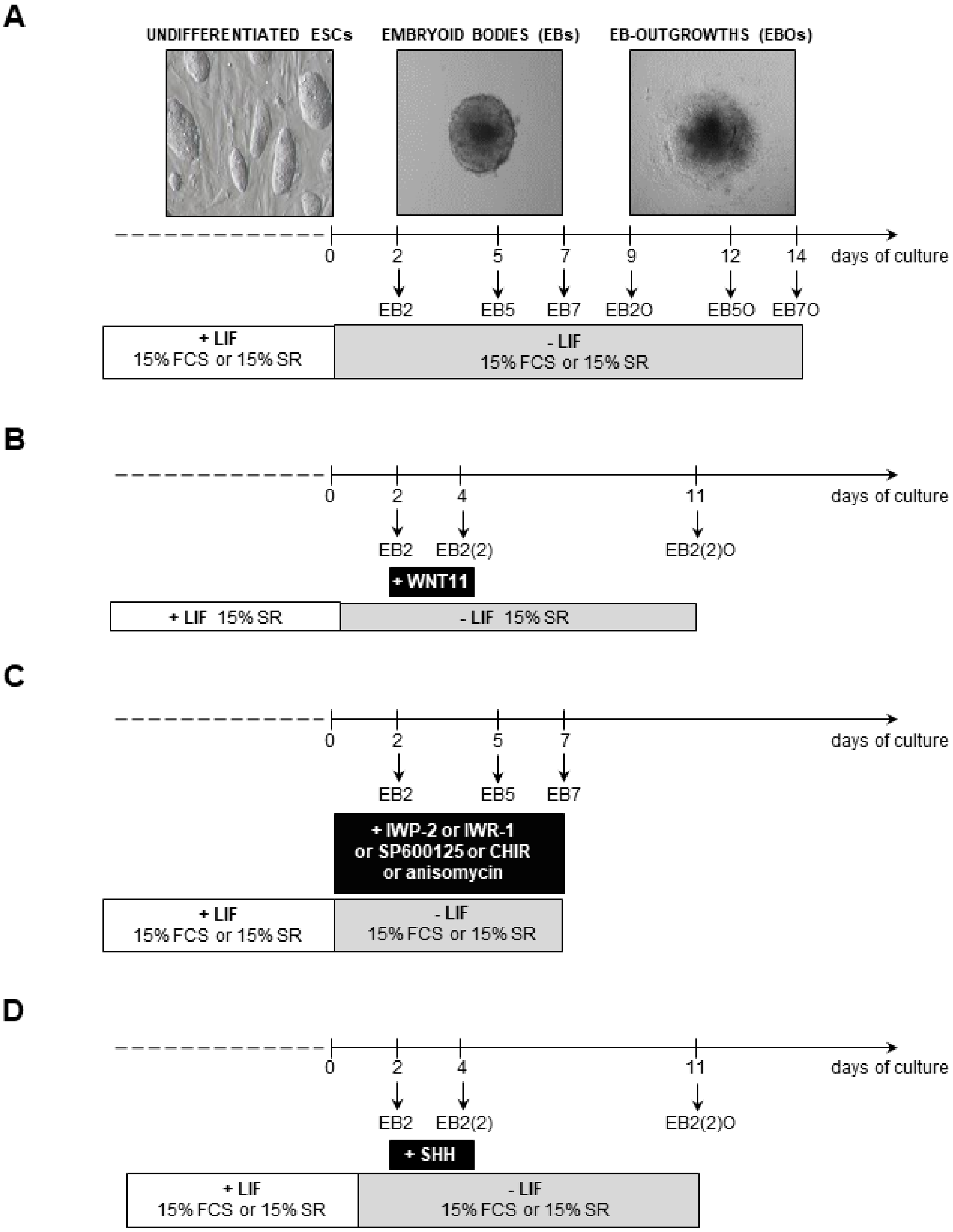

Figure S1. Outline of experiments.

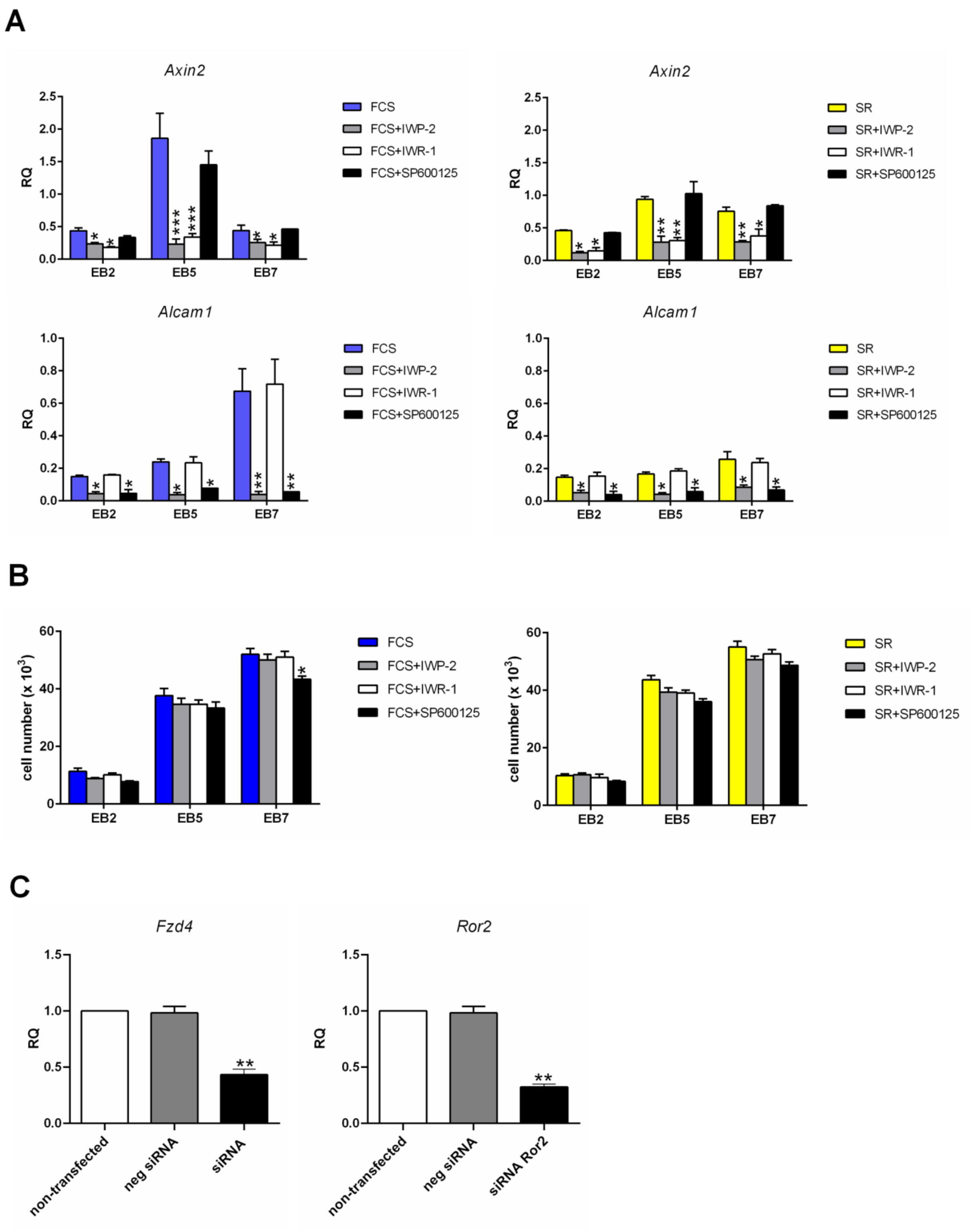

Figure S2. Influence of Wnt inhibitor or siRNA treatment on ESCs. (A) Expression of *Axin2* and *Alcam1*, targets of canonical and noncanonical Wnt pathways, in control EBs and EBs treated with indicated Wnt inhibitors. (B) Number of live cells counted after trypan blue staining building control EBs and EBs treated with indicated Wnt inhibitors. (C) Expression of *Fzd4* and *Ror2*, receptors for Wnt5a activating canonical and noncanonical Wnt pathway, in non-transfected or siRNA treated ECSs cultured in FCS. For A and C *β-actin* was used as a reference gene. RQ=1 for the expression level detected in 13.5-day old mouse embryo. Data presented as means of three independent experiments with standard deviations; *p<0.05; **p<0.01; ***p<0.001; ****p<0.0001.

Table S1. Taqman assays used in qPCR analyzes.

Gen	Taqman Assay ID	Gen	Taqman Assay ID
ActB	Mm01205647_g1	*Axin2*	Mm001443610_m1
Wnt1	Mm01300555_g1	*Alcam1*	Mm00711623_m1
Wnt2a	Mm00470018_m1	*Ror2*	Mm00443470_m1
Wnt2b	Mm00437330_m1	*Shh*	Mm00436528_m1
Wnt3	Mm00437336_m1	*Ptc1*	Mm00436026_m1
Wnt3a	Mm00437337_m1	*Ptc2*	Mm00436047_m1
Wnt4	Mm01194003_m1	*Ccnd1*	Mm00432359_m1
Wnt5a	Mm00437347_m1	*Gli1*	Mm00494654_m1
Wnt5b	Mm01183986_m1	*Nanog*	Mm02019550_s1
Wnt6	Mm00437347_m1	*Sox2*	Mm03053810_s1
Wnt7a	Mm00437356_m1	*Gata4*	Mm00484689_m1
Wnt7b	Mm01301717_m1	*Foxa2*	Mm01976556_s1
Wnt8a	Mm01157914_g1	*Pax6*	Mm00443081_m1
Wnt8b	Mm00442108_g1	*Tbxt*	Mm01318252_m1
Wnt9a	Mm00460518_m1	*Mixl1*	Mm00489085_m1
Wnt9b	Mm00457102_m1	*Msgn1*	Mm00490407_s1
Wnt10a	Mm00437325_m1	*Afp*	Mm00431715_m1
Wnt10b	Mm00442104_m1	*Dcx*	Mm00438400_m1
Wnt11	Mm00437328_m1	*Pdx1*	Mm00435565_m1
Wnt16	Mm00446420_m1	*TnnT2*	Mm01290256_m1
Fzd5	Mm01261677_g1	*Axin2*	Mm001443610_m1
Fzd7	Mm00433409_s1	*Alcam1*	Mm00711623_m1
